# Y chromosome-linked variation affects locomotor activity in male *Drosophila melanogaster* and is robust to differences in thermal environment

**DOI:** 10.1038/s41437-023-00604-x

**Published:** 2023-03-13

**Authors:** Sean Layh, Venkatesh Nagarajan-Radha, Bernardo Lemos, Damian K. Dowling

**Affiliations:** 1 School of Biological Sciences, Monash University, Melbourne, VIC Australia; 2 Behaviour Ecology and Evolution Lab, School of Life and Environmental Sciences, The University of Sydney, Camperdown, NSW Australia; 3 Molecular and Integrative Physiological Sciences Program, Department of Environmental Health, Harvard TH Chan School of Public Health, Boston, MA USA

**Keywords:** Evolution, Genetics

## Abstract

Although containing genes important for sex determination, genetic variation within the Y chromosome was traditionally predicted to contribute little to the expression of sexually dimorphic traits. This prediction was shaped by the assumption that the chromosome harbours few protein-coding genes, and that capacity for Y-linked variation to shape adaptation would be hindered by the chromosome’s lack of recombination and holandric inheritance. Consequently, most studies exploring the genotypic contributions to sexually dimorphic traits have focused on the autosomes and X chromosome. Yet, several studies have now demonstrated that the Y chromosome harbours variation affecting male fitness, moderating the expression of hundreds of genes across the nuclear genome. Furthermore, emerging results have shown that expression of this Y-linked variation may be sensitive to environmental heterogeneity, leading to the prediction that Y-mediated gene-by-environment interactions will shape the expression of sexually dimorphic phenotypes. We tested this prediction, investigating whether genetic variation across six distinct Y chromosome haplotypes affects the expression of locomotor activity, at each of two temperatures (20 and 28 °C) in male fruit flies (*Drosophila melanogaster*). Locomotor activity is a sexually dimorphic trait in this species, previously demonstrated to be under intralocus sexual conflict. We demonstrate Y haplotype effects on male locomotor activity, but the rank order and magnitude of these effects were unaltered by differences in temperature. Our study contributes to a growing number of studies demonstrating Y-linked effects moderating expression of traits evolving under sexually antagonistic selection, suggesting a role for the Y chromosome in shaping outcomes of sexual conflict.

## Introduction

The optimal expression of life history traits can differ substantially between the sexes. Yet, paradoxically, the genes that underpin many sexually dimorphic traits are shared by both males and females. Sexually antagonistic selection at shared loci can occur when alleles that differ in their fitness effects between the sexes compete with one another for optimal expression and transmission in each sex (Cox and Calsbeek 2009; Rice 1984). In cases of intralocus conflict, a genetic ‘tug-of-war’ between alleles with sex-specific effects can lead to an overall dampening of the optimal response to selection in each sex, a process that prevents either sex from evolving toward its trait optima (Bonduriansky and Chenoweth 2009). The evolution of sexual dimorphism is thought to represent a resolution to such intralocus sexual conflict, with sex chromosomes facilitating dimorphic trait expression by acting as repositories for genes with sex-specific expression (Bonduriansky and Rowe 2005; Griffin et al. 2013; Lande 1980; Poissant et al. 2010).

Research on the role of sex-chromosome linkage in moderating patterns of sexually dimorphic expression has traditionally focused on the chromosome found in each sex; the X chromosome in XY systems (Gibson et al. 2002; Rice 1984; Rice 2013; Rice and Chippindale 2001; Saifi and Chandra 1999; Tower 2006; Xirocostas et al. 2020). The Y chromosome has been less studied, presumably because it harbours very few protein-coding genes, lacks recombination, and is haploid, which in theory may reduce the efficacy of selection in shaping the genome and lead to heightened degradation of the genomic content of the chromosome (Bachtrog 2008; Engelstadter 2008), and make the Y more susceptible to the negative effects of Hill-Robertson interference (Bachtrog 2013; Hill and Robertson 1966). For example, in *Drosophila melanogaster*, the Y chromosome contains just 16 genes in total, despite the length of this chromosome comprising around 13% of the male genome (Zhang et al. 2020). Each of these genes is expressed exclusively during sex determination and spermatogenesis (Branco et al. 2013; Carvalho et al. 2009; Rohmer et al. 2004) and, furthermore, the chromosome harbours very low levels of segregating polymorphisms (Larracuente and Clark 2013). The remaining regions of the Y chromosome are entirely composed of densely packed non-coding heterochromatin (Chang and Larracuente 2019; Hoskins et al. 2002).

However, counter to traditional assumptions, research over the past decade has increasingly uncovered instances of Y-linked variation for male life history trait expression and fitness (Ågren et al. 2020; Archer et al. 2017; Chippindale and Rice 2001; Griffin et al. 2015; Jiang et al. 2010; Kaufmann et al. 2021; Kutch and Fedorka 2015; Kutch and Fedorka 2018; Lemos et al. 2010). Considerable molecular variation capable of trans-regulating hundreds, if not thousands, of autosomal and X-linked genes has been reported in non-protein-coding regions of the Y chromosome (Lemos et al. 2008). Other studies have indicated that such Y-linked regulatory variation is capable of influencing the expression of many sexually dimorphic traits including male reproductive success and fertility (Archer et al. 2017; Chippindale and Rice 2001; Yee et al. 2015; Zhang et al. 2000), suppression of sex-ratio distortion (Branco et al. 2013; Carvalho et al. 1997; Montchamp-Moreau et al. 2001), immunity (Kutch and Fedorka 2015), longevity (Griffin et al. 2015), and locomotor activity (Dean et al. 2015).

Furthermore, the results of several studies over the past two decades suggest that Y-linked variation could play a role in the regulation of sex-specific responses to environmental change, and moderate levels of plasticity in male responses to environmental change (David et al. 2005; Lemos et al. 2008; Rohmer et al. 2004), thereby shaping patterns of sexual dimorphism across environmental gradients. Notwithstanding, the pervasiveness of environment-mediated plasticity of Y chromosome variation remains generally understudied. Earlier work determined that the capacity of males to recover from heat-induced male sterility mapped to the Y chromosome (Rohmer et al. 2004; David et al. 2005), and that nuclear genes that are regulated by Y-linked variation are more sensitive to heat shock than other genes (Lemos et al. 2008). More recently, Dean et al. (2015) reported differences in male locomotor activity across strains of *D. melanogaster* that differed in their Y chromosome haplotype, with the rank order of performance of each of two haplotypes reversing according to whether flies were fed nutrient-rich or nutrient-deprived diets. Moreover, these nutrition-dependent differences in the pattern of Y-linked effects were only detected when flies were sampled under environments perceived as social (i.e., with exposure to pheromones of other males) rather than under solitary conditions. This suggests that Y chromosomes may harbour cryptic genetic variation that is uncovered only under exposure to environmental stress (McGuigan and Sgrò 2009), given that a perceived social environment will likely have triggered pre-copulatory behaviours mediated by intrasexual selection (Bretman et al. 2009).

Here, we further investigate the role of Y-linked variation in *D. melanogaster*, exploring the capacity for Y haplotype-by-environment interactions to affect male phenotypic expression. We focus on Y-linked plasticity that may be moderated by differences in the thermal environment, given that *D. melanogaster* is a cosmopolitan species with widespread global distribution and has evolved to tolerate considerable heterogeneity in thermal environments. For example, numerous studies have identified signatures of adaptive evolution along latitudinal clines that covary consistently with surface air temperatures (Camus et al. 2017; Hoffmann et al. 2002; Mettler et al. 1977; Sgrò et al. 2010). We focused on male locomotor activity, extending from the study of context-dependency in Y-mediated effects for this same trait previously identified by Dean et al. (2015) using two Y-haplotypes (Dahomey and Madang). Here, we extended sampling of Y-variation effects on locomotor activity to six distinct, and naturally-occurring haplotypes. The assays were replicated across two thermal environments, 20° and 28 °C, representing cold and warm environments for the flies that were otherwise reared in a common garden 25 °C environment. We were particularly interested in the measurement of locomotor activity because it is a sexually dimorphic trait in *D. melanogaster* with a genetic architecture that is largely shared across the sexes, as indicated by a high intersexual genetic correlation (Long and Rice 2007). It has also been shown to be under intralocus sexual conflict, with genotypes that are associated with high male, but low female fitness or vice versa (Long and Rice 2007). Therefore, Y-linked variation for this trait may evolve, in theory, to moderate levels of sexual dimorphism and outcomes of intralocus conflict over this trait.

## Methods

### Genetic panel of six Y chromosome haplotypes

Our experiment utilised a panel of genetic strains of *D. melanogaster* that differ only in their Y chromosome haplotype. The panel consisted of six different haplotypes, each placed alongside a standard genetic background. Each Y chromosome strain was created in replicate; i.e., two independent copies of each Y chromosome haplotype were created. The Y haplotypes were derived from isofemale lines originally sampled from six geographically distinct populations. These populations were BROWNSVILLE, Texas, USA, from David Rand (Rand et al. 1994); DAHOMEY, now Benin, Africa, derived from a massbred population collected in 1970; ISRAEL (ISR), eastern Mediterranean Israel, an isofemale wild type line derived from Bloomington Stock Centre; MADANG, Papua-New Guinea, derived from massbred stock (Kennington et al. 2001); PUERTO MONTT, Chile, isofemale derived from massbred population (Calboli et al. 2003); and ZIMBABWE, Central Southern Africa, Zim53 from JWO Ballard (Ballard and Kreitman 1994). We received each of these isofemale lines from David Clancy (now at University of Lancaster) in 2009, and propagated them in the laboratory to 2013, at which point we created each of the Y chromosome strains. Each of these six Y haplotypes was placed alongside a standardised nuclear background and common cytoplasmic background sourced from the *w*^*111*8^ nuclear background (Bloomington Stock number 5905) using a “chromosome replacement” crossing scheme described in Lemos et al. (2008) and Dean et al. (2015). Prior to the crosses, this *w*^*1118*^ strain had been subject to a protocol of inbreeding, with each generation propagated by a solitary full-sibling pair, over 10 s of generations, to achieve near-isogeneity (Innocenti et al. 2011). Moreover, before commencing the backcrossing scheme, each Y haplotype and the *w*^*1118*^ strain was treated with antibiotics (0.3 mg/ml tetracycline hydrochloride) to eliminate the intracellular bacterial symbiont, *Wolbachia*. This step was implemented for two reasons: to remove the potential for cytoplasmic incompatibility to interfere with the generation of the Y chromosome strains, and to remove the potential for any differences in the *Wolbachia* infection status across the strains to shape differences in locomotor activity of the strains.

To commence the crossing scheme, in the first generation males from the target isofemale lines (the Y donors) were crossed to females of a near-isogenic strain (Bloomington stock n. 4361) that contains recessive markers on all four of its chromosomes; yellow [*y*^*1*^ X chromosome], brown [*bw*^*1*^, 2nd chromosome], ebony [*e*^*4*^, 3rd chromosome], and cubitus interruptus and eyeless [*ci*^*1*^, *ey*^*R*^, 4th chromosome]. Sons of this cross were themselves crossed to females of stock 4361. Leveraging the lack of recombination in male *D. melanogaster*, we could determine that all chromosomes associated with the donor isofemale lines had been fully replaced by those of stock number 4361 by checking for the expression of each recessive phenotype. At this stage, our six target Y chromosome haplotypes sat alongside the nuclear and cytoplasmic background of the 4361 strain, each in two biological replicates. Males of each of these biological replicates were then independently backcrossed to females of the *w*^*1118*^ strain over four generations to replace the 4361 nuclear chromosomes with those of the *w*^*1118*^, again relying on the absence of recombination in males of this species. At this point, we ensured that chromosome replacement had been effective; only males first crossed to 4361 females and shown to not produce any progeny expressing any of the recessive markers were used to establish the Y lines.

Once the 12 strains were created, each was propagated by 10 flies (5 of each sex) per vial, across each generation, with no admixture between replicates of a given strain. All flies were provided with 6 ml of fresh standard laboratory food (made of dried potato mash, dextrose, yeast and 1% agar added with 10% Nipagin), supplemented with ad libitum dry activated yeast, and kept in 20 ml vials at 25 °C, and 12: 12 h day: night circadian cycle, unless stated otherwise below. At every generation, 10 new adult flies (5 per sex, 4-days post-eclosion into adulthood) were sampled per vial, and allowed to oviposit in these vials for 24 h, after which the adults were discarded. This propagation protocol continued for 3 years (2013–2016) prior to the experiment commencing. Several generations prior to the commencement of the experiment, we further partitioned each of the 12 strains into another tier of replication, keeping each strain across two vials, thus maintaining 24 “strain replicates” (each of six Y haplotypes existing across four replicates). We note that while our final panel of six haplotypes is smaller than some panels utilised to examine the question of Y-linked variation for male fitness traits, a strength of our panel was that each of the haplotypes was independently replicated (created and maintained long-term in two copies, and then further split into four copies prior to experiments); this provided high power to map effects to the level of the Y chromosome by partitioning out confounding sources of variance, such as residual and cryptic variation that may have built up in the autosomes, or other environmental sources of variation such as those associated with flies of the same genotype sharing the same environments (for example, vials) across time.

### Propagating experimental flies for locomotor activity

Our assays were designed to be replicated across eight experimental sampling days. We designed a breeding scheme that would enable us to collect age-matched focal males (flies used for assaying locomotor activity) harbouring each of the six Y chromosome haplotypes, on each sampling day of the assay. To achieve this, in the generation preceding the collection of the focal flies, we collected 10 flies (5 per sex) from each of the 24 strain replicates, and allowed them to oviposit over a 15 day period, transferring them into fresh vials every 24 h, and trimming the number of eggs per vial to 80 using a sterile spatula. The flies laying these eggs (i.e., the parents of the focal flies) were four days old post-eclosion on the first day of this ovipositing period, and 18 days of age on the 15^th^ day. We collected eggs from these ovipositing vials from parents aged 4, 5, 7, 8, 14, 15, 17 and 18 days of age, to produce focal flies for the experimental assay. This procedure allowed us to standardise the age of focal flies, but introduced a parental age effect of eight levels, which we included as a covariate in the statistical analysis.

We allowed the flies in each ovipositing vial to develop from egg to adulthood over a period of 10 days, under standard conditions, and then collected ten virgin males from each of the ovipositing vials (24 strain replicates × 8 days = 192 vials) within 6 h of their eclosion from pupae to adults, and under light CO_2_ anaesthesia. The ten virgin males collected per ovipositing vial were split into two groups of five males and housed in separate vials with access to fresh food (but no supplemented dry yeast) for 48 h. This resulted in 384 vials (48 per experimental sampling day) in total, designated as “vial ID” in our analysis. When 2 days of adult age, the males of each vial ID were moved to vials without food substrate for 48 h (the substrate consisted of agar only), since a previous study had established that this period of food deprivation increased subsequent locomotor activity of flies under the conditions of our assay (Dean et al. 2015). The focal flies were four days of adult age when measured for locomotor activity.

### Locomotor activity assay across two thermal environments

**Fig. 1:**
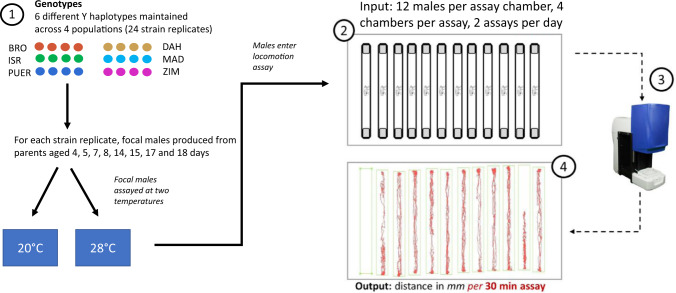
Experimental workflow for locomotor activity assay. 1. Each assay consisted of age standardised focal male flies of 24 different strain replicates (six Y chromosome haplotypes × 4 populations) produced by parents of eight different ages (4 to 18 days old). Focal males were isolated in polycarbonate tubes, and assigned to a 20° or 28 °C temperature treatment. 2. and 3. Focal males of each treatment were randomly allocated positions across four assay chambers, with 12 males loaded into each chamber, and a total of 48 males per assay. 4. Overall distance travelled by individual flies was then recorded in *mm*. Each assay was repeated twice per experimental day, across 8 sampling days. Ambient temperature treatment (either 20° or 28°) was alternated each day.

We assayed focal males for their total locomotor activity (overall distance travelled in mm) for a duration of 30 min using an automated motion-capture tracking system consisting of four temperature and light controlled assay chambers (“Zebraboxes”, Viewpoint Life Sciences, Lissieu, France), each having the capacity to simultaneously track the locomotor distance travelled by individual flies (Fig. 1). We designed our assays to sample total locomotor activity from two 4-day old males from each Vial ID, across two assays (denoted “assay number” in the statistical analyses) per day (one male per Vial ID per assay number), one immediately following the other, with both assays being conducted within the first 90 min of the commencement of the flies’ daylight-cycle (lab controlled 8 AM AEST). We restricted assays to two per day, both in early to mid-morning, to minimise any circadian effects on the activity phenotype of the flies (Nagarajan-Radha et al. 2020). Each assay consisted of sampling 48 focal flies across four assay chambers (12 per chamber), noting that some flies were subsequently lost during handling (more details provided below).

The flies were assayed in complete darkness to restrict their capacity to interact using visual cues with other flies within the same chamber. Prior to each assay, we transferred individual flies, via aspiration (without the use of anaesthesia), into new and previously unused polycarbonate tubes (65 × 5 mm, Trikinetics, USA), and plugged both ends with coarsely cut foam stoppers. Each tube housed one focal fly. The available space for fly movement within the tube was standardised to 45 mm in length and 5 mm in width. Tubes were placed equidistant from each other within a custom-engineered plastic frame and placed inside one of the four assay chambers. We balanced each assay by having two flies from each Y-haplotype, each from a different strain replicate, in each assay chamber (12 flies per chamber). The positions of these flies within each chambers were then randomised across each assay via a random number generator, so that flies of a given strain replicate were not assayed in the same position within the chamber over multiple assays during the experiment.

**Fig. 2:**
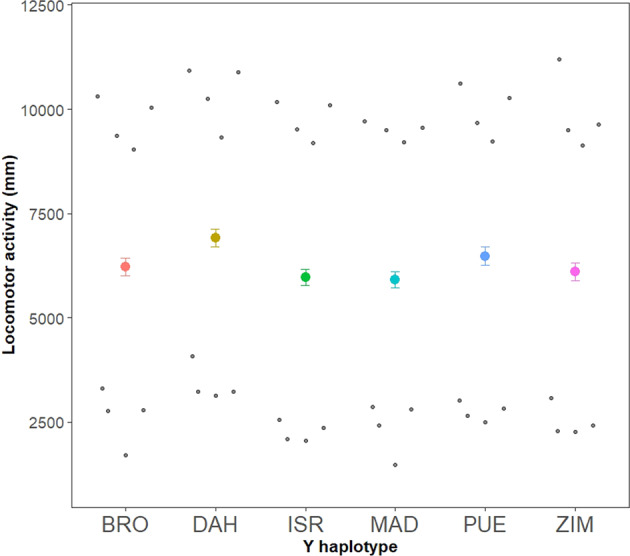
Estimated Marginal Means (±1 standard error) for locomotor activity (total distance in mm travelled in 30 min) of males across six distinct Y-chromosome haplotypes of *D. melanogaster*. Labels on the horizontal axis indicate the origin population of each Y haplotype: BRO = Brownsville (TX, USA), DAH = Dahomey (Benin, Africa), ISR = Israel, MAD = Madang (Papua-New Guinea) PUE = Puerto Montt (Chile) and ZIM = Zimbabwe. Sample sizes per haplotype were BRO = 103, DAH = 91, ISR = 116, MAD = 121, PUE = 94, ZIM = 106. EM means ± 95% Confidence Intervals for each haplotype are BRO = 6219 ± 448, DAH = 6910 ± 453, ISR = 5972 ± 426, MAD = 5907 ± 422, PUE = 6479 ± 453, ZIM = 6111 ± 445). Data clouds in background represent the means of raw data associated with each combination of strain replicate (4) × temperature (2) for each Y haplotype (8 datapoints per Y haplotype); datapoints above the mean values of each Y haplotype come from the 28 °C temperature treatment, and those below the mean values come from the 20 °C treatment. The large additive effect of temperature is evident here, where the warmer assay temperature was associated with much higher activity, with a mean >9000 mm, relative to the cooler assay temperature with a mean <3000 mm.

We replicated the assays across two temperatures, 20° and 28 °C. The temperature within the assay chambers was maintained via a central heating/cooling system and was simultaneously monitored using temperature probes placed inside each of the assay chambers. We alternated the two temperature treatments, 20° and 28 °C, across each experimental sampling day, such that focal flies whose parents were 4, 7, 16 and 19 days of age were measured at 20 °C, and those with parents of 5, 8, 15 and 18 days were measured at 28 °C. In total, we sought to assay 768 flies (across 384 vials), but inadvertent losses of some flies during handling, and technical glitches in the automated analysis of the locomotor data whereby the software failed to track a fly throughout the entire duration of the assay, reduced our final dataset to 631 flies (across 337 vials). The total number of flies sampled per Y haplotype is reported in Fig. 2.

### Statistical analyses

We calculated the total locomotor activity (mm) of each individual male fly using Viewpoint Zebralab software v3.22 (ViewPoint Life Sciences). Data analysis was conducted in R-studio (version 1.4.1717) with a linear multilevel (mixed effects) model in the lme4 package (Bates et al. 2012). Total locomotor activity was modelled as the response variable, with Y haplotype (6 levels), temperature (2 levels), assay chamber (4 levels), and assay number (2 levels) as fixed effects and parental age as a covariate. We tested for interactions between these five parameters. We also included strain replicate (24 levels), and vial ID (337 levels) as random intercepts in our analysis to account for the hierarchical structure of the dataset. Y haplotype effects were estimated at the level of the strain replicate (i.e., the error degrees of freedom, or units of replication, reflect the number of strain replicates = 24 levels), and all other effects and interactions at the level of the vial ID (337 levels) (Arnqvist 2020). Modelling Y haplotype effects at the level of the strain replicate provided us with a means to statistically partition Y-linked variation from other residual genetic variation that may have existed across the strain replicates, and from environmental sources of variance that flies of a given strain replicate or vial ID would share.

We then employed a stepwise elimination method to simplify the full model by removing non-significant (*p* > 0.05) higher-order interactions, using the *drop1* function and *F tests* with Kenward-Rogers approximation of degrees of freedom. We derived a final model using this approach, from which we determined the parameter estimates for each fixed effect using restricted maximum likelihood method of estimation and Type III F tests with Kenward-Rogers approximation of degrees of freedom in the *car* package (Fox and Weisberg 2011), and sum to zero contrasts. The variance attributed to each random effect, and the residual variance, was estimated using restricted maximum likelihood (REML), and is denoted in Standard Deviation.

## Results

The locomotor activity of adult males was affected by Y chromosome haplotype (Table 1). In particular, males carrying the Y haplotype derived from Dahomey were more active than males of any other haplotype (Table 2, Fig. 2). Although males were much more active when assayed at the warmer temperature (males moved an average of 9.8 m across the 30 min assay when tested at 28 °C, and 2.6 m at 20 °C), there was no interaction between Y chromosome haplotype and temperature on locomotor activity (F = 0.67, *p* = 0.65), and the interaction was therefore dropped from the final model.

**Table 1:** Results of a linear multilevel (mixed effects) model.

**Fixed effects**	***F***_df(num,den)_	***P***
Intercept	5411.15_1, 16.23_	
Y-haplotype	3.17_5, 16.02_	0.035
Temperature	3000.02_1, 300.99_	<0.001
Parental age	25.23_1,318.82_	<0.001
Assay chamber	4.47_3,68.96_	0.006
Assay number	10.11_1, 319.18_	0.002
Temperature × Parental age	16.96_1, 305.11_	<0.001
Assay chamber × Parental age	5.41_3,307.12_	0.001
Assay number × Parental age	6.22_1, 320.97_	0.013
**Random effects**	**SD**	
Strain replicate	254.5	
Vial ID	640.2	
Residual	1385.9	

Total locomotor activity of each individual fly was used as the response variable in the model. Y-haplotype, assay temperature, assay chamber and assay number were modelled as fixed effect factors, and parental age as a covariate and mean-centred. Factors explaining the hierarchical structure of the dataset—strain replicate (*n* = 24 levels, which represented the number of populations used to estimate the error degrees of freedom when estimating the Y-haplotype effect) and vial ID (*n* = 337 levels) were modelled as random intercepts. Fixed effect parameter estimates were determined using restricted maximum likelihood method of estimation, and Type III F tests with Kenward-Rogers approximation of degrees of freedom and sum-to-zero contrasts, in the car package (Fox and Weisberg 2011). Standard deviation (SD) associated with each random intercept, and the Residual SD, is shown.

**Table 2:** Parameter estimates of six Y-haplotypes, as estimated from the linear multilevel model in Table 1, using sum-to-zero contrasts in which estimates are relative to the global mean.

Effect	Estimate	SE	*t* value	*p*
REFERENCE	6266.31	84.92	73.794	<0.00001
BROWNSVILLE	−47.04	191.17	−0.246	0.809408
DAHOMEY	643.88	195.26	3.298	0.004851
ISRAEL	−294.76	183.26	−1.608	0.133185
MADANG	−358.98	181.83	−1.974	0.072065
PUERTO MONT	212.7	195.08	1.09	0.292945
ZIMBABWE	−155.8	189.57	−0.822	0.425996

**Fig. 3:**
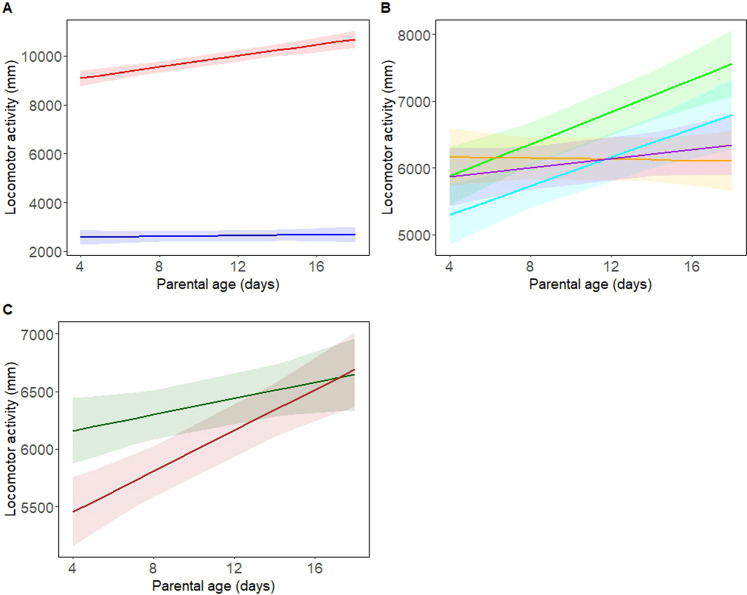
The relationship between parental age and locomotor activity of the focal flies was shaped by interactions with temperature, assay chamber and assay number. **A** Linear regression lines (and 95% confidence intervals) between parental age and locomotor activity at 20° (denoted in blue) and 28° (red). **B** Regression lines (and 95% CIs) between parental age and locomotor activity across the four assay chambers (chamber 1 in light green, 2 in light blue, 3 in orange, 4 in purple). **C** Regression lines (and 95% CIs) between parental age and locomotor activity across assay number (assay number 1 in dark green, 2 in brown).

The age of the parents affected the locomotor activity of males, with males produced by older parents generally exhibiting higher locomotor activity. However, the slope of this relationship was contingent on interactions with three separate variables; firstly parental age interacted with temperature at which the focal males were assayed, whereby the positive association between parental age and locomotor activity was only observed when focal males were assayed at 28 °C (Fig. 3A), secondly parental age interacted with the assay chamber in which the focal males were measured (Fig. 3B), and thirdly parental age interacted with the assay number in which the focal males were measured, whereby the slope of the relationship between parental age and locomotor activity of the focal flies was steeper in the second assay of any given day (Fig. 3C).

## Discussion

The aims of this study were to investigate firstly whether genetic variation found across six distinct Y haplotypes affected locomotor activity of male *D. melanogaster*, and secondly to test whether any such Y-linked regulation of activity was sensitive to differences in the thermal environment. We found Y-linked effects on locomotor activity, although these effects were largely shaped by the effects of one haplotype in particular, sourced from Dahomey, which exhibited heightened locomotor activity relative to males harbouring any of the other five haplotypes. This result thus confirms the capacity for Y-linked variation to shape the locomotor activity of male fruit flies. Yet, although locomotor activity was highly sensitive to the thermal environment, the effects of the Y chromosome haplotypes on activity were not sensitive to the thermal environment, with no interaction between Y haplotype and temperature. These findings advance our understanding of the regulatory influence of the Y chromosome on expression of fitness-related traits that share a common genetic architecture across the sexes, by confirming the capacity for Y-linked variation to modify the expression of such traits, and potentially moderate the outcomes of intralocus sexual conflict over trait evolution. We discuss these insights below and suggest avenues for future research.

### Y-linked variation mediates male locomotor activity

Our results demonstrate that the Y chromosome harbours genetic variation that affects male locomotor activity. This observation, however, was specific to the Dahomey haplotype, which differed significantly from the others by conferring a heightened level of locomotor activity. Our study extended from an earlier study by Dean et al. (2015), who found context-dependent effects of the Y chromosome on locomotor activity, when studying effects across just two haplotypes, both of which were included in our study and one of which was the Dahomey haplotype. Thus, although we increased the number of Y haplotypes in our study from the two studied by Dean et al. (2015) to six, the effects we observed were nonetheless limited to the Dahomey haplotype.

This raises the question of how prevalent Y-linked effects on locomotor activity in particular, and male phenotypes in general, are in nature. It is possible that the Dahomey-specific effect was shaped by the cumulative effect of numerous polymorphisms specific to that Y haplotype, under an assumption that levels of nucleotide divergence between pairwise combinations of Y haplotypes would be associated with levels of phenotypic divergence in locomotor activity. The prediction here would be that nucleotide divergence would be greatest for pairwise combinations involving the Dahomey Y haplotype, which may then explain the Dahomey-specific effect on locomotor activity. While we do not possess the sequence data of the Y haplotypes in our study since the Y chromosome consists almost entirely of heterochromatic sequence (Chang and Larracuente 2019; Hoskins et al. 2002), published data exist for the corresponding mtDNA haplotypes isolated from the same isofemale lines from which the six Y haplotypes were sampled in this study. Flies sampled from Dahomey share a very similar mtDNA haplotype to those sampled from Madang and Zimbabwe (Camus et al. 2012; Wolff et al. 2016). Thus, if we were to assume that the phylogenetic relationships between Y chromosome haplotypes used in our study are similar to those of the mtDNA haplotypes across the same populations, then it might be expected that the locomotor activity phenotype of flies with the Dahomey haplotype would be similar to those with Madang and Zimbabwe haplotypes. This was not the case. Alternatively, it is possible that the Dahomey Y haplotype harbours a single rare polymorphism of major effect, which is either absent or segregating at low frequency in most populations of *D. melanogaster*. If so, this would suggest that the Y chromosome will have little effect on shaping the evolution of sexual dimorphism in locomotor activity in general within populations in this species. Testing between the above possibilities would require an experimental design that harnesses a greater number of Y haplotypes sourced from natural populations, to home in on representative levels of Y-linked variation for this trait (Chippindale and Rice 2001; Griffin et al. 2015; Kutch and Fedorka 2015). These data could ultimately be reconciled with deep sequencing data of the Y chromosome haplotypes, to test whether variation within heterochromatic areas of the Y chromosome may map to locomotor activity phenotypes.

Several lines of evidence, however, do suggest that Y-linked variation may routinely mediate expression of male phenotypes. Previous studies of Y-linked variation in *D. melanogaster* have shown widespread capacity of the Y to regulate 100 s of nuclear genes throughout the rest of the genome (Lemos et al. 2008), and affect components of male reproductive fitness (Chippindale and Rice 2001), lifespan (Griffin et al. 2015), locomotor activity (Dean et al. 2015) and immune response (Jiang et al. 2010; Kutch and Fedorka 2015). Furthermore, recent insights suggest that much of this response is likely to be epistatic, with the nature of the Y-linked effects determined by the genetic background that the Y haplotypes are assayed within. For example, Jiang et al. (2010) revealed that patterns of Y-linked regulatory variation on the expression of autosomal and X-linked genes are contingent on the genetic background of *D. melanogaster*; while Ågren et al. (2020) demonstrated that epistatic interactions between mtDNA and Y chromosome haplotype in *D. melanogaster* shaped the expression of hundreds of nuclear genes, with genes involved in male reproduction being most sensitive to such interactions.

When it comes to the expression of traits tied to male reproductive success, Yee et al. (2015) detected complex interactions between mtDNA haplotype, Y haplotype, and age on the proportion of male flies mating. Again, their study used three of the same Y haplotypes that we leveraged in our study (Dahomey, Israel and Madang), and found Y-effects that extended to other haplotypes and were not just specific to the Dahomey Y, but which were highly context dependent on both the mtDNA haplotype that the Y haplotype was assayed against, and also the age of the male flies assayed. Finally, a recent study by Archer et al. (2017) revealed that the effects of three distinct Y chromosome haplotypes on male offspring production in *D. simulans* were moderated by the genetic background of the flies. Clearly, therefore, Y-linked effects have capacity to be context-dependent in their expression, across different environmental contexts and different genetic backgrounds, and further research is required to elucidate the nature of this context-dependency and the evolutionary implications for sex-specific life-histories.

### Y-linked variation for locomotor activity is robust to differences in thermal environment

In light of the previously-reported context-dependency in the effects of Y-linked variation on expression in male phenotypes, we predicted that any Y-linked effects detected in our study would be contingent on the thermal environment in which the Y haplotypes were screened. This prediction was reinforced by the knowledge that the focal trait in our study—locomotor activity— is particularly sensitive to modifications to thermal environment (Kjaersgaard et al. 2010). However, we found no evidence for Y-mediated differences in levels of thermal plasticity for locomotor activity. Based on visual observation of datapoints of each of the six haplotypes across the two temperatures, and supported by the absence of a statistically significant interaction, there was no evidence for temperature-specific differences in the pattern of effects across Y haplotypes, with the Dahomey haplotype associated with the highest activity across both temperatures.

This lack of thermal sensitivity of the Y chromosome haplotypes is surprising, given that previous studies have shown that Y-linked variation modulates thermal sensitivity of spermatogenesis in response to temperature, and that genes affected by Y-linked variation are more responsive to heat shock than other genes (Lemos et al. 2008). Kjaersgaard et al. (2010) have demonstrated that correlated population-level responses in locomotor activity evolve in response to selection for extreme thermal stress (heat shock and cold shock) in *D. melanogaster*. The two temperatures that were utilised in our study (20 and 28 °C) fall within the natural range of temperatures experienced by *D. melanogaster* within its species distribution. We propose that it would be fruitful to further investigate the capacity for extreme thermal environments to uncover cryptic genetic variation for locomotor activity within the Y chromosome. Such a study could inform the capacity for the Y chromosome to contribute to adaptive responses to environmental extremes under projected scenarios of climate change.

### The potential for Y-linked variation to mediate outcomes of sexual conflict over optimal life histories

Y-linked variation clearly contributes to the expression of male-limited reproductive traits (Archer et al. 2017; Chippindale and Rice 2001; Yee et al. 2015). Yet, several studies over the past decade have now uncovered Y-linked variation shaping the expression of traits, including locomotor activity (Dean et al. 2015) and immune capacity (Kutch and Fedorka 2015), which share a common genetic basis between the sexes and that are likely to be characterised by strong positive intersexual genetic correlations. These Y-linked effects therefore have the potential to shape sexual dimorphism in these traits, through trans-regulation of the nuclear genes that encode these traits. Furthermore, because the genes that encode these traits are shared across the sexes, their evolutionary trajectories are constrained under intralocus conflict between the sexes over which alleles should be transmitted across generations. Indeed, Long et al. (2007) confirmed that locomotor activity in *D. melanogaster* is underpinned by a strongly positive intersexual genetic correlation, and shaped by sexually antagonistic selection—although their study did not seek to examine whether any of the genetic variation attributable to locomotor activity mapped to the Y chromosome. Our results provide a proof-of-concept for the capacity for the Y chromosome to moderate the outcomes of trait evolution under intralocus conflict. We believe this contention warrants future research attention, particularly in light of a recent series of studies that indicate that a common set of nuclear genes is co-regulated not only by paternally-transmitted Y chromosome, but by maternally-inherited mtDNA genome (Ågren et al. 2020; Rogell et al. 2014), suggesting a large fraction of the transcriptome might be at the centre of an evolutionary tug-of-war between the sexes, regulated by genomic regions exhibiting sex-limited transmission.

### Plasticity of locomotory activity in response to complex environmental variation

Although it was not a primary aim of our study, our design enabled us to model the effects of a number of intrinsic and extrinsic environmental factors on male locomotor activity. We observed a high degree of plasticity of the trait in response to variation in these factors, with locomotor activity highly sensitive to the environmental temperature of the assay, the parental age of the focal flies, as well as the identity of the assay chamber and the assay number in which the focal flies were measured. Effects of temperature on activity have previously been observed (Kjaersgaard et al. 2010), and are not surprising given well established effects of temperature on metabolic rate in insects (Gillooly et al. 2001). However, the effects of the other environmental effects were unexpected, and represent fine scale responses to environmental heterogeneity experienced by the flies during these experiments. The parental age effects we identified on locomotory activity were particularly intriguing; focal males born to increasingly older parents exhibited higher locomotor activity. Yet, both the magnitude and direction of this association was moderated by interactions with the assay temperature, with the direct assay environment, and with the time of measurement of the focal flies. These results add to growing evidence that indirect transgenerational effects may routinely interact with direct environmental effects to shape patterns of phenotypic variation (Bonduriansky 2021). Understanding the ecological and evolutionary implications of these complex parental-by-offspring environmental effects on phenotypic expression remains a challenge for the future.

## Conclusion

We found Y-linked effects on locomotor activity of male *D. melanogaster*, although these effects were limited to one particular Y haplotype conferring higher activity than others. These effects were largely independent of differences in temperature, suggesting Y-linked moderation of locomotor activity may be robust to changes in the thermal environment. Taken together, the findings suggest the contribution of the Y chromosome to male fitness extends beyond the contributions to sex determination and spermatogenesis that the chromosome was traditionally believed to be limited to, moderating expression of male phenotypes that are core to organismal performance, and possibly evolving under sexually antagonistic selection. We encourage future research to further explore the dynamics of Y-linked moderation of male-specific life histories, through investigation of the range of trait types affected, the degree of context-dependency in effects of Y-linked variation, and the speed and evolutionary lability by which Y chromosomes can respond to sexually antagonistic selection and mediate trajectories of male trait evolution.

## Data Availability

Data available at https://figshare.com/s/7a4ab422c8c0e48c248f.
